# Roles of microRNAs and Long Non-Coding RNAs Encoded by Parasitic Helminths in Human Carcinogenesis

**DOI:** 10.3390/ijms23158173

**Published:** 2022-07-25

**Authors:** Ana Gabriela Leija-Montoya, Javier González-Ramírez, Gustavo Martínez-Coronilla, María Esther Mejía-León, Mario Isiordia-Espinoza, Fausto Sánchez-Muñoz, Elda Georgina Chávez-Cortez, Viviana Pitones-Rubio, Nicolas Serafín-Higuera

**Affiliations:** 1 Facultad de Medicina Mexicali, Universidad Autónoma de Baja California, Centro Cívico, Mexicali 21000, BC, Mexico; gabriela.leija@uabc.edu.mx (A.G.L.-M.); gustavoj@uabc.edu.mx (G.M.-C.); esther.mejia86@uabc.edu.mx (M.E.M.-L.); 2 Facultad de Enfermería, Universidad Autónoma de Baja California, Av. Álvaro Obregón y Calle “G” S/N, Col. Nueva, Mexicali 21100, BC, Mexico; javier.gonzalez.ramirez@uabc.edu.mx; 3 Instituto de Investigación en Ciencias Médicas, Departamento de Clínicas, División de Ciencias Biomédicas, Centro Universitario de Los Altos, Universidad de Guadalajara, Av. Rafael Casillas Aceves 1200, Tepatitlán de Morelos 47600, JAL, Mexico; mario.isiordia162@yahoo.com; 4 Departamento de Inmunología, Instituto Nacional de Cardiología, Juan Badiano No. 1, Col. Sección XVI, Tlapan 140080, DF, Mexico; sanchezmunozfausto@gmail.com; 5 Centro de Ciencias de la Salud, Facultad de Odontología, Universidad Autónoma de Baja California, Zotoluca s/n, Fracc. Calafia, Mexicali 21040, BC, Mexico; chavez.elda@uabc.edu.mx (E.G.C.-C.); viviana.pitones@uabc.edu.mx (V.P.-R.)

**Keywords:** microRNA, long non-coding RNA, carcinogenic parasite, cancer, helminths, infections, inflammation, fibrosis, polarization of immune cells

## Abstract

Infectious agents such as viruses, bacteria, and parasites can lead to cancer development. Infection with the helminthic parasite *Schistosoma haematobium* can cause cancer of the urinary bladder in humans, and infection with the parasites *Clonorchis sinensis* and *Opisthorchis viverrini* can promote cholangiocarcinoma. These three pathogens have been categorized as “group 1: carcinogenic to humans” by the International Agency for Research on Cancer (IARC). Additionally, the parasite *Schistosoma japonicum* has been associated with liver and colorectal cancer and classified as “group 2B: possibly carcinogenic to humans”. These parasites express regulatory non-coding RNAs as microRNAs (miRNAs) and long non-coding RNAs (lncRNAs), which modulate genic expression in different biological processes. In this review, we discuss the potential roles of miRNAS and lncRNAs encoded by helminthic parasites that are classified by the IARC as carcinogenic and possibly carcinogenic to humans. The miRNAs of these parasites may be involved in carcinogenesis by modulating the biological functions of the pathogen and the host and by altering microenvironments prone to tumor growth. miRNAs were identified in different host fluids. Additionally, some miRNAs showed direct antitumoral effects. Together, these miRNAs show potential for use in future therapeutic and diagnostic applications. LncRNAs have been less studied in these parasites, and their biological effects in the parasite–host interaction are largely unknown.

## 1. Introduction

RNA was long considered to only be a link between DNA and proteins; it was given a secondary role in protein production [1]. However, this idea had to be reconsidered after more than three-quarters of the human genome was found to be able to be transcribed into RNA, with only 2% ultimately coding for proteins. These non-coding RNAs (ncRNAs) have been shown to be of great importance in cellular activities and individual health [2,3].

Essentially, ncRNAs can be divided, based on their length, into long ncRNAs (>200 nt) and small ncRNAs (<200 nt) [4]. Long non-coding RNAs (lncRNAs) are a class of ncRNA molecules with various functions such as gene regulation, dosage compensation, and epigenetic regulation. It has been observed that the dysregulation and genomic variations in several lncRNAs can be associated with the development of diseases. It should also be noted that lncRNAs can be viable indicators of the physiological status of cells by presenting tissue- and developmental-specific expression [5]. On the other hand, small RNAs are just as diverse, but better-characterized; in this way, several small RNAs with different functions are recognized [6]. Within the small RNAs, we find microRNAs (miRNAs), RNAs that interact with Piwi (piRNAs), small interfering RNAs (siRNAs), small nuclear RNAs (snRNAs), small nucleolar RNAs (snoRNAs), small cytoplasmic RNAs (scRNAs), transfer RNAs (tRNAs), ribosomal RNAs (rRNAs) [7], and, of those whose importance has been found more recently, circular RNAs (circRNAs) [8].

According to the World Health Organization (WHO), during 2019, cancer was ranked as the first or second cause of death in 112 countries worldwide, and the third or fourth in another 23 countries. In addition to this, the leading diseases in mortality, such as cerebrovascular accidents and coronary diseases, have shown a decrease, while cancer is becoming the main disease that limits life expectancy [9]. Cancer is highly complex, and new, distinctive characteristics of cancer have emerged, such as phenotypic plasticity and interrupted differentiation, in addition to the presence of nonmutational epigenetic reprogramming and polymorphic microbiomes. These characteristics allow the determination of the distinctive capacities of different types of cancer [10].

**Figure 1:**
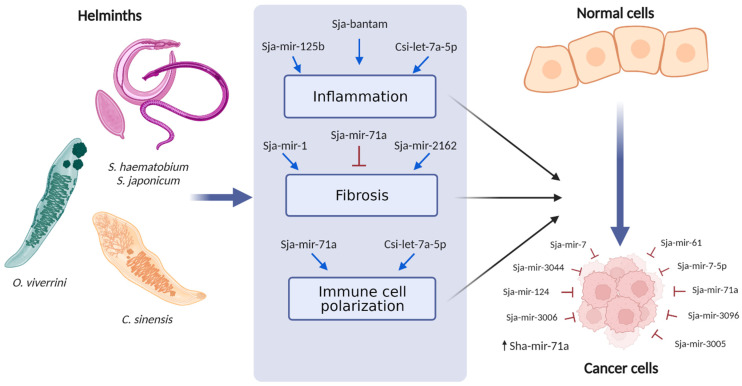
Helminths may promote tumorigenesis through different processes such as chronic inflammation; the polarization of immune cells such as macrophages and T cells; or by inducing persistent injury in the tissues, which can lead to undesirable effects such as fibrosis. miRNAs expressed and secreted by helminths involved in human cancer can modulate these processes. Some helminth miRNAs have shown direct antitumoral activity. miRNAs from parasites could be useful in therapy and diagnostics; for example, an miRNA of *S. haematobium* (Sha-mir-71a) is abundant in the urine of patients with bladder cancer associated with infection. Created using BioRender.com.

In this context, infections with viruses and bacteria have been recognized for many years as being associated with human carcinogenesis [11]. It should be added that many tropical parasitic infections remain a public-health problem in medicine, and some of these are proven to be related to carcinogenesis. Thus, it has been proven that the etiopathogenesis of many cancers is directly related to parasitic infections. Well-known tropical parasitic infections that can induce carcinogenesis include opisthorchiasis, clonorchiasis, and schistosomiasis [12]. In this review, we describe the lncRNAs and microRNAs of helminthic parasites that are classified by the International Agency for Research on Cancer (AICR) as group 1: carcinogens in humans (*Schistosoma haematobium*, *Clonorchis sinensis*, and *Opisthorchis viverrini*) [13] and group 2: possible carcinogens in humans (*Schistosoma japonicum*) [14] and discuss their possible roles in cancer (Figure 1).

In this narrative review, PUBMED was searched in May 2022 using the terms: microRNA, long non-coding RNA, cancer, carcinogenic parasite, helminths, schistosome, clonorchis, and opisthorchis using the Boolean operators “AND” and “OR”. Articles in English were included. Reports of miRNAs and lncRNAs encoded by helminths that are involved in human carcinogenesis (classified by the AICR as groups 1 and 2) were included. Thus, reports of miRNAs and lncRNAs of helminths such as *S. mansoni*, *S. intercalatum*, *S. mekongi,* or *O. felineus* (not classified by the AICR as group 1 or 2) were not included. Reports of miRNAs and lncRNAs encoded by hosts and involved in cancers that are associated with infection with helminths were not included.

## 2. microRNAs (miRNAs)

miRNAs are small non-coding RNAs of ~22 nt, which can be found in animals, plants, and some viruses regulating the expression of an enormous number of genes [15]. Their expressional profiles are distinct in different tissues [16]. Most miRNAs can be transcribed by RNA polymerase II or III, generating primary precursor miRNAs (pri-miRNAs) [17], which can be processed through the canonical or non-canonical pathways [18]. miRNAs can be intragenic or intergenic based on their position throughout the genome. Intragenic miRNAs are localized within genes in exonic or intronic regions. Additionally, miRNAs can be organized as single RNAs or as groups of RNAs (clusters). miRNA clusters can be transcribed polycistronically, which allows the coordinated regulation of complex biological processes and functional redundancy in some cases [18]. Generally, miRNAs bind the 3′-(UTR) untranslated region of messenger RNA (mRNA) to suppress expression [15], but they can also bind to the 5′-UTR region of mRNA to stimulate gene expression [16]. miRNAs perform their functions mainly in the cytoplasm; however, miRNAs also can be localized in other compartments such as the nucleus, mitochondria, endoplasmic reticulum, and Golgi apparatus [18].

It has been proposed that miRNAs are involved in communication between cells. Donor cells can transfer miRNAs through gap junctions to neighbor cells. Additionally, miRNAs can be released into the circulation via extracellular vesicles, apoptotic bodies, binding HDL (high-density lipoprotein), or RBP (RNA-binding proteins) [19].

miRNAs participate in the maintenance of relevant processes such as cellular metabolism, development, immunity, and growth. However, aberrant expressions of miRNAs in human cancers have been widely reported. Different miRNAs have been implicated in cancer progression, proliferation, metastasis, angiogenesis, tumor-associated inflammation, modulation of the tumor microenvironment, and the response of tumor cells to chemotherapy [15,16].

Reports of miRNAs that were analyzed in a cancer context have resulted in the concept of tumor-suppressor miRNAs, which are downregulated in cancer in which their target oncogenes are overexpressed. On the other hand, oncomiRs have higher expression in tumors and inhibit the expression of target tumor-suppressor genes [19]. However, the functions of particular miRNAs in cancer can become more complex, presenting dual functions [16].

Changes in host miRNA expressional profiles during the course of infectious diseases have been widely reported [18,20]; these changes have been associated with impaired host biological functions. The miRNAs of parasites also change their expression patterns in concordance with the relevant biological processes of the pathogens, and they are influenced by the host microenvironment. The major miRNAs of pathogens regulating critical functions, such as spreading, chronic infection, inflammation, development, or reproduction, could be the targets of potential new drugs.

miRNAs have been detected in different bodily fluids, which suggests significant potential, such as their use as biomarkers in diagnostics and prognostics. Additionally, these versatile regulatory molecules could be used in therapeutic applications, although this potential will be explored and determined in future studies [16,21].

## 3. Long Non-Coding RNAs (lncRNAs)

Imprinted Maternally Expressed Transcript (H19) and X-Inactive-Specific Transcript (Xist) were among the first lncRNAs to be described and characterized, as well as among the first to have a discovered function. However, until then, it was thought that their regulatory function was an exception instead of a regulatory mechanism that the cell uses constantly and permanently [22].

lncRNAs are RNAs that have a diverse genomic location since they can be produced from regions as diverse as enhancer regions, intergenic regions, and introns, and can even derive from antisense RNAs [23]; in terms of the characteristics of the elements that make them up, they have a very similar structure to messenger RNAs since many have a cap at their 5′ end; moreover, they are subjected to the elimination of introns and the union of exons, in addition to presenting a poly-A tail [24]. Regarding their cellular location, stability, conservation, and production specificity in tissues, lncRNAs can be found in the nucleus and the cytoplasm, and their half-life is variable, ranging from about 2 h to 16 h on average. Additionally, their sequence of nucleotides is very little-conserved between species; they are produced in all tissues, but are especially abundant in the brain and the central nervous system [25].

In terms of their functions and mechanisms of action, lncRNAs can bind to DNA, other RNAs, and even proteins [26]; they can serve as signaling molecules, decoys, guides, and scaffolds for DNA-binding proteins [27]. In relation to their influence on the regulation of protein production, they can change the patterns of chromatin organization and activate or repress the activation of genes; moreover, they act as competitive endogenous RNAs (ceRNAs) and can also participate in the modification of mRNA and proteins, among others [23,28].

In this sense, due to the wide range of interactions and functions of lncRNAs, it is not surprising that they can coordinate several physiological processes and that their dysfunction can be involved in various human diseases. Thus, to date, potential mechanisms of lncRNAs that can regulate gene expression and associations with diseases, such as cancer, have been found. Further studies on cancer associated with pathogens will help us to better understand the possible regulations of lncRNAs and potential therapeutic targets [29].

## 4. Helminths Involved in Cancer

Helminths include free-living and parasitic Platyhelminthes (flatworms) and Nematoda (roundworms) [30]. Even though both phyla infect people, and some of them are related to cancer development, only three Platyhelminthes are classified by the IARC as carcinogenic in humans. The IARC evaluates the carcinogenic risk of agents and classifies them into four groups: group 1: the agent is carcinogenic in humans; group 2: the agent is probably (2A) or possibly (2B) carcinogenic in humans; group 3: the agent is not classifiable as to its carcinogenicity to humans; and group 4: the agent is probably not carcinogenic in humans [14]. Platyhelminthes include cestodes and trematodes, also known as flukes. The flukes classified in AIRC group 1 are *Schistosoma haematobium*, *Clonorchis sinensis*, and *Opisthorchis viverrini*, which are associated with urine bladder, bile duct, and liver carcinoma; *Schistosoma japonicum* is classified in group 2B as possibly carcinogenic in humans, and *S. mansoni* is classified in group 3 [13,14].

As mentioned, some helminth species are known to be involved in human cancer development [30], and the initial evidence of their mechanism of involvement pointed to chronic inflammation as the common factor in the initiation and development of cancer. Indeed, oncogene activation, suppressor-gene inactivation, and somatic mutations were described as important processes in the initiation and promotion of malignancy [31,32,33,34,35,36]. Additional studies proposed that helminth metabolites acting as genotoxins or growth factors were involved in the development of cancer initiation through genetic mutation and angiogenesis, respectively. Further studies have explored the microbiome role in carcinogenesis and have demonstrated that chronic infection by carcinogenic helminths can lead to changes in the microbial communities, enlarging the inflammatory and fibrotic responses related to cancer development [30]. Even though recent studies support that helminth carcinogenesis, as with most cancers, is likely to be initiated by a biological or chemical stimulus, followed by chronic inflammation, fibrosis, and alterations in the cellular microenvironment, the exact mechanisms or molecules used by the carcinogenic species of helminths are still not fully understood [30,31,32,33,34,35,36].

### 4.1. Schistosoma Haematobium and Schistosoma Japonicum

*S. haematobium* is a trematode that lives in pairs and undergoes sexual reproduction. Humans are infected via skin penetration of the free-swimming parasite in freshwater environments [37]. *S. haematobium* chronic infection leads to squamous-cell carcinoma (SCC) and urothelial carcinoma of the urinary bladder [38]. To infect the host, the fluke penetrates the skin via the secretion of proteolytic enzymes. Then, the worms move through the circulation to the site of infection such as the bladder, uterus, or prostate for reproduction. When the females release eggs, some of them are excreted in the urine to continue their life cycle, but the remaining eggs are deposited in the bladder; these will cause damage and inflammation to the bladder lumen [39], increasing the risk of bladder cancer. Urogenital schistosomiasis (UGS) is a chronic inflammation-mediated disease. The continuous inflammatory reaction to the eggs leads to parenchymal tissue destruction, fibrosis, granulomata, and ultimately, to fibrotic nodules termed sandy patches [38,40]. The eggs retained due to chronic infection release H03-H-IPSE, a major ortholog of the interleukin-4-inducing principle (IPSE); this nuclear protein is related to urothelial cell proliferation in mouse models. Additionally, it induces bladder angiogenesis and allows the eggs to escape the host immune response, leading to hyperplasia [37,39]. Adult flukes also increase cell proliferation and migration as they decrease apoptosis. Chronic infection must be present for the development of carcinogenesis, as a single exposure to a fluke antigen will not contribute to cholangiocarcinoma (CCA). Moreover, bacterial and fluke co-infection has been reported to increase bladder cancer risk [37]. This is supported by reports of urine microbiome dysbiosis in urogenital schistosomiasis in bladder cancer and other pathologies of the organ [41]. Additionally, fluke infection and exposure to carcinogenic agents such as smoking or N-nitroso compounds increase bladder cancer risk [37,42,43]. *S. haematobium* infections are associated with activation-loss of p53, retinoblastoma (RB), anti-apoptotic pathways [37,44], and epigenetic changes leading to hyperplasia and cancer. Urogenital schistosomiasis can also cause molecular perturbation via overexpression of the fibroblast growth factor receptor protein 3, leading to aggressive cell proliferation. In addition, mutations in KRAS have also been observed [45].

*S. japonicum* has been associated with liver and colorectal cancer [46]. Epidemiological studies have supported this association. Additionally, pathological alterations are mainly associated with retained and deposited eggs, promoting an inflammatory reaction, continuous irritation, granuloma formation, and microabscesses, which could induce fibrosis and hyperplasia [46]. Chronic inflammation, damage to DNA, and molecules released by parasites have been associated with the promotion of cancer [46].

### 4.2. Clonorchis Sinensis

*C. sinensis* are thin and translucent worms; the adult flukes are elongated and dorsoventrally flat, 10–25 mm long, and 1.5–4.0 mm wide [47]. The first intermediate hosts of *C. sinensis* are species of freshwater snails, and fish are the second intermediate hosts. Humans become infected through encysted metacercariae consumption contained in raw or improperly cooked fish [48]. *C. sinensis* is related to the incidence of CCA and was classified by the IARC as a definitive biological carcinogenic agent in 2009 [49]. Briefly, the development of CCA requires a sequence of events: pathogen infection, chronic inflammation, wound healing, cellular proliferation, genetic/epigenetic mutations, and malignant transformation [47]. In humans, after a month of infection, the adult worms develop in the biliary ducts. Then, the wall of the bile duct becomes vulnerable to mechanical irritation and the bile may be biochemically altered by the adult fluke; these events are key factors in tissue inflammation and its transition to CCA [31]. Although the mechanisms of the development of hyperplasia are not well-understood, *C. sinensis* promotes an inflammatory response around the biliary tract and could trigger changes in the epithelium lining the bile duct through a stage of severe hyperplasia to dysplasia. Then, the proliferative ducts become susceptible to tumor initiation by carcinogens, even in small doses, that are not related to cancer development in non-infected individuals [31]. Additionally, it was reported, in an in vitro study, that excretory–secretory (ES) products may promote carcinogenesis via a synergic effect, even when using small amounts of the carcinogenic compounds [31]. Furthermore, ES products are related to free-radical generation through the activation of Toll-like receptor isoforms and the NF-kB pathway, contributing to chronic inflammation. ES products are also related to metastasis, morphological cell changes, malignant transformation, and more aggressive phenotypes of CCA cells [50,51,52,53]. Metaplasia of biliary epithelial cells into mucin-producing goblet cells increases mucin production and creates gland-like areas in the mucosa; these areas may become encased by fibrous tissue as a result of chronic infection and could lead to cholangiofibrosis. The development of CCA has been observed in the presence of adenomatous changes in the wall of the bile duct [31]. Together, the pathological mechanisms of CCA associated with *C. sinensis* include three main factors: mechanical injury of the biliary epithelia by the fluke, inflammation-associated immunopathological changes followed by secondary infection, and the effects of ES products [47,54].

### 4.3. Opisthorchis Viverrini

*O. viverrini* is a leaf-shaped worm of 8–12 mm in length; like *C. sinensis*, it has two intermediate hosts before infecting humans: the first intermediate host is a snail, and the second host is a fish. Humans acquire metacercariae of *O. viverrini* by eating raw or poorly cooked fish; after a month, the adult fluke matures and lives in the biliary tract, producing mechanical irritation and changes in the ductal epithelium. *O. viverrini* reside in the large and medium-sized bile ducts, but, in cases of heavy infection, the liver flukes can reach the gallbladder, the common bile duct, and the pancreatic duct [31]. Infection leads to dilatation of the bile ducts with fibrosis, hyperplasia, desquamation, and adenomatous proliferation and infiltration by lymphocytes, monocytes, eosinophils, and plasma cells. The inflammatory response and ES products have a key role in carcinogenesis. *O. viverrini* produces an inflammatory response using a pathway that results in inducible nitric oxide synthase and ciclooxygenase-2. Free radicals resulting from the immune response can induce damage to the host tissue, leading to DNA damage and mutations. Whole-exome and targeted sequencing has confirmed mutations in CCA-related genes such as TP53, KRAS, SMAD4, and genes associated with chromatin remodeling, WNT signaling, and KRAS/G protein signaling [55]. Briefly, *O. viverrini* infection may promote cholangiocarcinoma development through DNA damage induced by iNOS expression from inflammatory cells and the epithelium of bile ducts [31]. Additionally, an in vitro assay demonstrated that ES products stimulate pRB and cyclin D1, allowing the cell cycle to continue and proliferate. Evidence in animal models indicated that the RB pathway may be involved in CCA development related to *O. viverrini* infection. Furthermore, Ov-GRN-1 is a growth factor with sequence similarity to the mammalian growth factor, granulin. Ov-GRN-1 is present in the *O. viverrini* ES products of adult flukes and induces host-cell proliferation, establishing the flukes’ role in creating a tumorigenic environment [55]. Together, *O. viverrini* leads to CCA via three possible pathways: mechanical damage by the fluke; immunopathology due to reactive oxygen intermediates (ROI) and nitric oxide (NO); and the direct effect of ES products inducing cell proliferation and inhibiting DNA repair/apoptosis [55], suggesting the possibility of regulatory functions in the host; however, this has not been corroborated experimentally.

## 5. miRNAs of *Schistosoma haematobium*

It was predicted that 89 microRNAs were encoded and expressed by adult *S. haematobium* worms [56]. However, a recent in silico analysis identified 149 mature miRNAs and 131 precursor miRNAs, mainly in intergenic regions [57]. Considering the seed sequence of miRNAs from *S. haematobium*, homology with *S. mansoni* and *S. japonicum* in 43 miRNAs was described [56]; additionally, 34 miRNAs specific to *S. haematobium* were predicted [56]. Additionally, another study reported 41 conserved miRNAs in the *Schistosoma* genus [57]. Interestingly, 64 miRNAs were differentially expressed in male and female adults of *S. haematobium,* suggesting possible functional roles in reproductive biology [56].

The five top-ranked miRNAs expressed in *S. haematobium* include Sha-mir-1, Sha-mir-71a, Sha-mir-125b, Sha-mir-7a, and Sha-mir-let7, which have homology with miRNA seeds from *S. mansoni* and *S. japonicum* [56]. Although possible targets of these *S. haematobium* miRNAs have been predicted [56], their biological functions in worms, host infection, bladder cancer, and vesicular transport have not yet been addressed experimentally. Additionally, the identification of schistosomal miRNAs (bantam and mir-2c-p3) in the extracellular vesicles of host biological fluids such as serum has been proposed as a diagnostic tool by which to detect infection with *S. haematobium* [58] and possibly prevent infection-associated cancer; thus, the utility of Sha-mir-71 as a biomarker in bladder cancer has been proposed [59].

### Sha-miR-71a

Sha-mir-71a is an expected miRNA of 23 nucleotides in length and is highly expressed in male and female adults of *S. haematobium* worms [56]. It was predicted that Sha-mir-71a can bind 3′-UTR elements of 53 host genes [56], suggesting the possibility of regulatory functions in the host; however, this has not been corroborated experimentally.

Recently, the expression of Sha-mir-71a was analyzed in the urine samples of patients with bladder cancer to assess its potential as a biomarker [59]. Sha-mir-71a was abundantly found in the urine of patients with bladder cancer as compared to benign bladder cystitis associated with schistosomiasis (Figure 1). Additionally, this miRNA was more highly detected in urine samples from patients with bilharzial bladder cancer than bladder cancer not associated with bilharziasis (schistosomiasis), suggesting its specificity in the identification of bladder cancer associated with infection [59]. Sha-mir-71a expression was found to be distinct in different bladder cancer types, being overexpressed in transitional-cell carcinoma in comparison with squamous-cell carcinoma [59]. This is interesting since the identification of the histological variants and subtypes of bladder cancer can have an impact on prognosis and therapy [60,61]; thus, the stratification of the groups of the study can provide more valuable information. It has been suggested that Sha-mir-71a could be a biomarker with diagnostic and prognostic value [59]; however, its biological function in *S. haematobium* and its role in bladder cancer have not been described. Further studies are necessary to describe the role of miRNAs expressed by *S. haematobium* in the promotion of cancer and their possible applications as therapeutic targets or biomarkers.

## 6. LncRNAs of *Schistosoma haematobium*

A bioinformatics analysis predicted 3589 lncRNA transcripts in the eggs, adult males, and adult females of *S. haematobium* [62]. However, the characterization and differential expression of the lncRNAs in the distinct development stages of *S. haematobium* have not been described yet. Interestingly, homology between 694 lncRNAs from *S. haematobium* and *S. mansoni* was identified, which could assist in defining the possible functions of *S. haematobium* lncRNAs [62]; further studies must address the molecular mechanisms of lncRNAs encoded by *S. haematobium* that are involved in development, sexual maturation, egg production, packaging in extracellular vesicles, the regulation of targets in the host, relationships with inflammation, fibrosis, and cancer.

## 7. miRNAs of *Schistosoma japonicum*

### 7.1. miRNAs in Development and Sexual Maturation

Different reports have identified miRNAs and predicted their possible molecular targets, their organization in the genome (as the presence of clusters), and their expression in the different developmental stages of *S. japonicum* [63,64,65,66,67,68,69,70,71]. Additionally, miRNA expression profiles were found to differ between male and female worms [63]. Recently, an analysis of *S. japonicum*’s known 79 miRNAs at different numbers of days post-infection aimed to determine the dynamics of miRNA expression during pairing, maturation, and egg production, and identified three clusters in each gender [64]. The cluster 1 grouped miRNAs were highly expressed during paring; the cluster 2 grouped miRNAs were highly expressed during development and sexual maturation; and the cluster 3 grouped miRNAs were highly expressed in the egg-production stage. An in silico analysis predicted different biological functions for each cluster [64]. These reports suggest that specific miRNAs could have particular functions across the lifespan of *S. japonicum* and that changes in the expression patterns of miRNAs could be a response to the exposition of *S. japonicum* to different microenvironments during its life cycle. It is possible that miRNAs expressed in specific stages could be potential targets for use in therapy in the future, or as biomarkers to prevent infection or adverse effects such as chronic inflammation, which has been associated with cancer.

The sexual maturation of female worms and egg production are key events in the dissemination and pathogenesis of schistosomiasis, with the pairing of male and female worms being a precondition for these events [72]. Thus, female worms originating from single-sex infection (infection with exclusively female cercariae) showed defects in egg production and development, as well as a reduced damage potential to the host [73]. These underdeveloped female worms presented differential expressions of miRNAs as compared with totally mature females (originating from double-sex infection) [73,74,75]. Moreover, different studies have determined that the miRNA expression landscapes were distinct in male and female schistosomulum (before pairing) and adult male and female worms (after pairing), indicating their involvement in sexual development [67,74,76].

The differential expression of 38 miRNAs by *S. japonicum* male and female worms during pairing, gametogenesis, and the production of eggs was reported [72]. Of these, 14 miRNAs predominated in male worms and 4 miRNAs were favorably expressed in females, validating some molecular targets of these miRNAs. These results suggest the capacity of these miRNAs to modulate the expression and possibly regulate the sexual maturation and development of *S. japonicum* [72]. Moreover, Sja-bantam, Sja-mir-31, and Sja-mir-750, expressed preferentially in the ovaries of the worms, were found to be essential in the maintenance of ovarian architecture and oocyte maturation. Importantly, Sja-mir-750 expression also allowed the maintenance of egg production [72,77], which is important in chronicity, pathogenesis, and the spread of the infection.

Paired adult worms can release extracellular vesicles with miRNA cargo, which can be internalized by the host cells [78,79]. In addition to extracellular vesicles, it was suggested that miRNAs can be secreted in complexes with proteins such as Argonaute 2/3 [79]. Interestingly, adult male and female worms can release extracellular vesicles with different profiles of the miRNA cargo [77]. Importantly, Sja-mir-3479 and Sja-bantam were found to be more expressed in the extracellular vesicles of paired worms than in male-only or female-only extracellular vesicles, suggesting that pairing modulates the expression of miRNAs [79]. Furthermore, stimuli such as the ingestion of erythrocytes and heme proteins promote sexual maturation and egg production, and it was reported that these stimuli also increased the expression of Sja-mir-3479 and Sja-bantam in the extracellular vesicles of paired worms and female-only worms [79]. The inhibition of calpain, an enzyme involved in the shedding of microvesicles, via treatment with calpeptin in pairing worms reduced the production of Sja-mir-3479 and Sja-bantam in extracellular vesicles and egg production. Thus, miRNAs packaged in extracellular vesicles could participate in communication during pairing and regulate the maturation and fecundity of *S. japonicum* [79].

Different miRNAs are expressed by *S. japonicum* when the parasites are recovered from hosts that are more susceptible or less susceptible to infection [80,81,82,83]. It has been described that species less susceptible to *S. japonicum* infection, such as water buffalos (*Bubalus bubalis*), rats (*Rattus norvegicus*), reed voles (*Microtus fortis*), and immunodeficient mice (severe combined immunodeficiency (SCID) and nude mice), do not offer appropriate conditions for processes such as the growth, development, sexual maturation, and egg production of this parasite, resulting in the failure of the parasite to complete its life cycle. Thus, comparative analyses with susceptible hosts such as mice (*Mus musculus*) or yellow cattle (*Bos taurus*) have allowed researchers to determine the participation of miRNAs in these essential biological phenomena [80,81,82,83]. These reports show that the distinct components and characteristics of the microenvironment provided by the host can impact the miRNA expression profiles of *S. japonicum*. It is possible that miRNAs participate in crosstalk between the host cells and the different developmental stages of the parasite. Studies aimed at identifying the molecules or precise conditions that generate *S. japonicum* miRNA expression profiles, such as the profiles reported in less susceptible hosts, could be useful in treatments.

Together, these observations indicate that different miRNAs are very important in the development and sexual maturation of male and female parasites, and could participate in communication during pairing and support the establishment and spread of the infection. Thus, they could be used as targets in therapies at different stages to prevent chronic inflammation, fibrosis, or detrimental effects that could be associated with the development of cancer.

Chronic infection with *S. japonicum* can induce hepatic granulomatous inflammation, fibrosis, and hepatosplenomegaly; the release of soluble egg-derived antigens, including extracellular vesicles, have an important role in the generation of these signs [84,85]. Moreover, inflammation and fibrosis were associated with the promotion of cancer. Since schistosomal mature eggs are relevant in the pathology and prevalence of hepatosplenic schistosomiasis, miRNAs in this stage have previously been characterized [63,85]. It was suggested that certain families of miRNAs are expressed preferentially in different developmental stages of the parasite and that members of the Sja-mir-71 family are more expressed in the egg stage [85]. Additionally, an increased expression of Sja-bantam, Sja-mir-3479-3p, and Sja-mir-8185 in eggs, as compared to schistosomula and adult worms, has been described [86]. miRNAs from eggs have been shown to have immunomodulator effects and to participate in liver fibrosis, which is addressed in later sections. In this sense, miRNAs packaged in egg-extracellular vesicles, such as Sja-mir-71b and Sja-bantam, can be delivered to different host cells (for example, hepatocytes) and modulate gene expression [87].

### 7.2. miRNAs and Liver Fibrosis: Sja-mir-1, Sja-mir-2162, and Sja-mir-71a

**Figure 2:**
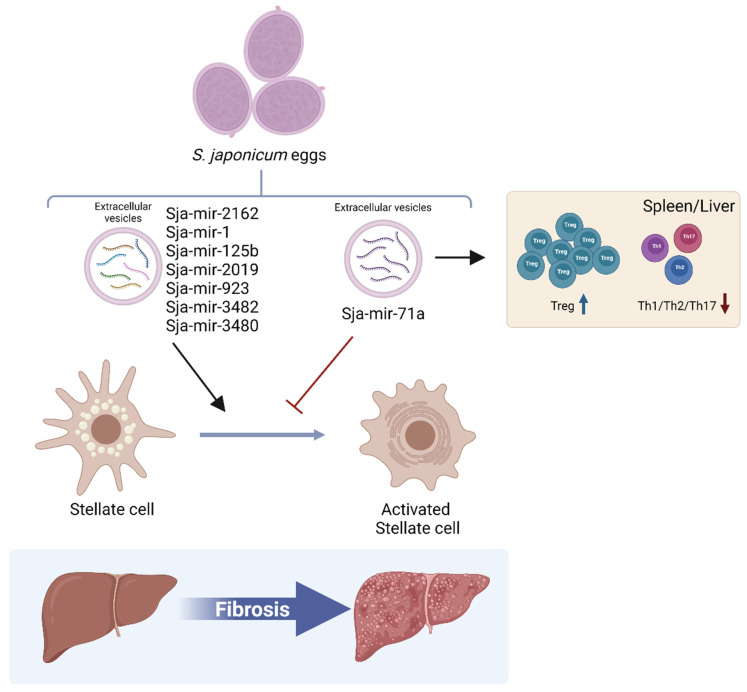
Eggs of *S. Japonicum* can release extracellular vesicles, which transport different miRNA cargo that can be internalized by host cells as liver stellate cells and exert distinct effects. These miRNAs can repress the genic expression of host-cell molecular targets, promoting the activation of liver stellate cells. This has been associated with the generation of liver fibrosis. In contrast, Sja-mir-71a can inhibit the activation of stellate cells and prevent fibrosis. Additionally, Sja-mir-71a induces a reduction in Th1, Th2, and Th17 cells in the liver and spleen, having immunomodulatory functions that possibly influence the characteristics of the microenvironment in host tissues. Created using BioRender.com.

*S. japonicum* eggs release extracellular vesicles that can carry different miRNA cargo such as Sja-mir-1, Sja-mir-2162, and Sja-mir-71a. These extracellular vesicles can be internalized by hepatic stellate cells (HSC) and delivery molecule cargo [84,88,89] (Figure 2).

Additionally, extracellular vesicles from *S. japonicum* can reach other host-cell types and tissues such as the heart, brain, liver, spleen, kidney, lung, and thymus [88] and possibly release their contents; for example, Sja-mir-2162 has been detected in Kupffer cells and hepatocytes [84]. Sja-mir-1, Sja-mir-2162, and Sja-mir-71a inside the HSC can bind different target molecules, inducing the degradation of mRNAs [84,88,89]. It was established that Sja-mir-1 and Sja-mir-2162 can induce hepatic fibrosis in vivo and promote the activation of HSC; this was represented by the increased expression of α-smooth muscle actin (α-Sma) and collagens in these cells [84,89]. However, the increased expression of Sja-mir-71a resulted in opposite effects both in vivo and in vitro; moreover, this miRNA presents antitumoral activity, as described in the following section [88] (Figure 2).

The described molecular mechanism of these miRNAs involves the binding of Sja-mir-1 to the 3′-UTR region of its molecular target, Sfrp1 (Secreted Frizzled-Related Protein 1), resulting in reduced expression of the product of this gene. SFRP1 inhibits the Wnt/Beta-Catenin pathway in HSC; however, Sja-mir-1 negatively regulates SFRP1 and promotes Wnt/Beta-Catenin signaling and the activation of HSC [89]. On the other hand, Sja-mir-2162 can bind the 3′-UTR region of the molecular target, transforming growth factor beta receptor III (tgfβ3) and decreasing the expression at the level of its mRNA and protein [84]. Since TGFβ3 can negatively regulate the TGFβ pathway, the presence of Sja-mir-2162 promotes TGFβ signaling and the phosphorylation of SMAD transcription factors, which can induce the expression of fibrotic genes [84]. Interestingly, the expression inhibition of Sja-mir-1 and Sja-mir-2162 was found to revert the observed effects, and their potential role in therapy has been suggested [84,89]. Examples of other miRNAs with the capacity to activate HSC, but without the molecular mechanisms described, include Sja-mir-125b, Sja-mir-219, Sja-mir-923, Sja-mir-3482, and Sja-mir-3480 [84]. Hepatic fibrosis and the activation of HSC could be involved in the promotion of tumorigenesis [90]; thus, further analyses of these described miRNAs could determine their functions or potential therapeutic effects in the context of cancer.

Regarding Sja-mir-71a, the proposed mechanism by which it exerts its effects implies attachment to the 3′-UTR region of Sema4d (Semaphorin 4D), resulting in the reduced expression of this target; this inhibits the TGβ1/SMAD and IL-13/STAT6 pathways in HSC and causes decreased fibrosis [88]. Together, these observations suggest a dual role of miRNAs from *S. japonicum* egg-extracellular vesicles in liver fibrosis, although it has been suggested that methodological differences in the isolation of extracellular vesicles could have generated discrepancies in the results [88].

### 7.3. miRNAs and Immunomodulation: Sja-mir-125b, Sja-bantam, and Sja-mir-71a

Extracellular vesicles released by adult *S. japonicum* worms can transport miRNAs such as Sja-bantam and Sja-mir-125b and can be delivered to host immune cells, being captured by monocytes, macrophages, T cells, B cells, and NK (natural killer) cells in vitro and during natural infection [91]. Inside immune cells such as macrophages, *S. japonicum* miRNAs can exert biological effects, inhibiting the expression of host molecular targets and promoting the proliferation of these cells [91].

**Figure 3:**
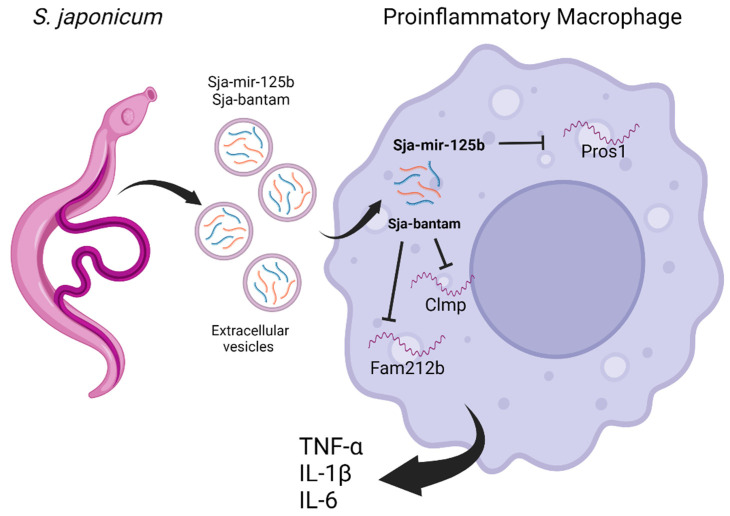
*S. japonicum* worms can release extracellular vesicles that transport miRNA cargo, such as Sja-mir-125b and Sja-bantam. These miRNAs are internalized by host macrophages, bind molecular targets, and induce a proinflammatory phenotype. Moreover, these miRNAs secreted by the worms promote the proliferation of macrophages. Created using BioRender.com.

Sja-mir-125b can bind the 3′-UTR region of the Pros1 (Protein S1) target, reducing its expression in macrophages. Pros1 participates in the inhibition of the Toll-like receptor pathways; however, its decrease results in an increased expression of proinflammatory molecules such as p38 mitogen-activated protein kinase, Traf5 (TNF receptor associated factor 5), Irf7 (interferon regulatory factor 7), IL-1β, IL-6, and TNF-α (tumor necrosis factor-alpha) [91]. The enhanced expression of these proinflammatory molecules can also be induced by Sja-bantam. The molecular mechanism involves the binding of this miRNA to Fam212b (Inka box actin regulator 2) and Clmp (CXADR-like membrane protein) host target mRNAs, decreasing their expression [91] (Figure 3).

It was suggested that the increase in the number of monocytes and the expression of TNF-α observed in infections by *S. japonicum* could be, in part, due to the effects exerted by Sja-mir-125b and Sja-bantam. Importantly, since an induced reduction in the number of macrophages during *S. japonicum* infection decreases the number of worms and eggs in the liver, Sja-mir-125b and Sja-bantam could be crucial in the promotion of parasite survival [91] and chronic infection. Furthermore, cytokines such as TNF-α can induce an inflammatory microenvironment, which has been associated with cancer promotion. Thus, it is possible that mir-125b and Sja-bantam indirectly encourage tumoral development by allowing chronic infection and inflammation. However, further studies should be performed due to the controversial role of *S. japonicum* in cancer.

Additionally, the overexpression of Sja-mir-71a was found to promote an increased number of regulatory T cells (Treg) and a reduced number of Th1, Th2, and Th17 cells in the spleen and liver of *S. japonicum*-infected mice; it was proposed that this could alleviate liver fibrosis [88]. Extracellular vesicles released by *S. japonicum* can be internalized by T cells [92], suggesting that mir-71a could be transported and delivered by extracellular vesicles to T cells, thereby inducing polarization. Thus, *S. japonicum* eggs can modulate the immune response in distant sites via miRNAs such as Sja-mir-71a (Figure 2). Potential therapeutic uses and the prevention of these miRNAs in a cancer context should be analyzed.

### 7.4. Antitumoral miRNAs: Sja-mir-61, Sja-mir-7-5p, Sja-mir-71a, and Sja-mir-3096

The overexpression of *S. japonicum* miRNAs with tumor-suppressor activity could function as potential therapy for cancer. It has been noted that Sja-mir-61 can inhibit the migration of liver tumor cells in vitro, and its molecular mechanism was found to involve the binding of Sja-mir-61 to the 3′-UTR region of oncogene phosphoglycerate mutase 1 (PGAM1), diminishing its expression [93]. Moreover, Sja-mir-61 was found to inhibit tumoral growth in a murine tumor model and showed antiangiogenic properties [93]. Sja-mir-7-5p is another miRNA with reported tumor-suppression activity and with a homologous miRNA in humans. Sja-mir-7-5p can induce the arrest of the cell cycle and inhibit the proliferation and migration of liver tumor cells in vitro, as well as tumoral growth in vivo. These effects are exerted by the interaction of the miRNA with the SKP2 (S-phase kinase-associated protein 2) gene, resulting in reduced genic expression of this target. This leads to an increase in the cyclin-dependent kinase inhibitor protein P27 and a decrease in the matrix metalloproteinase 9 protein, which are involved in cell-cycle arrest and cellular migration, respectively [94]. In addition to its immunomodulation and antifibrotic activities, Sja-mir-71a also inhibits the proliferation and migration of tumor cells through its interaction with the 3′-UTR region of the Frizzled Class Receptor 4 (FZD4) gene, reducing its expression. FZD4 is a receptor of Wnt signaling and it has been associated with the promotion of cancer [95]. Furthermore, it has been noted that Sja-mir-3096 can be packaged and transported in extracellular vesicles secreted by *S. japonicum* eggs and delivered to host liver cells. This miRNA can inhibit the proliferation and migration of tumor cells and suppress tumoral growth in vivo. To exert these effects, Sja-mir-3096 binds to the 3′-UTR region of the phosphoinositide 3-kinase class II alpha (PIK3C2A) gene, diminishing its expression and resulting in decreased phosphorylation of mTOR, a signaling component that promotes proliferation [96] (Table 1).

Other miRNAs with the capacity to inhibit the proliferation of tumor cells without molecular mechanisms describing their antitumoral activity include Sja-mir-7, Sja-miR-124, Sja-mir-3005, Sja-mir-3006, and Sja-mir-3044 [95,96]. Together, these observations reveal the therapeutic potential of *S. japonicum* miRNAs and the controversial role of *S. japonicum* in carcinogenesis. In this sense, the dual properties of cancer promotion and inhibition were suggested [96]. Additionally, these reports analyzed the antitumoral activities of miRNAs in liver tumor cells; it would be interesting to extend the knowledge of the possible therapeutic effects on others cancer types.

### 7.5. Circulating miRNAs in Host Serum/Plasma

In addition to describing the molecular mechanisms of *S. japonium*-derived miRNAs, different studies have described circulating miRNAs as having the potential to be used as a diagnostic tool for *S. japonicum* infection. Since *S. japonicum* extracellular vesicles have been detected in host serum, it is possible that circulating miRNAs detected in the host fluids are packaged in extracellular vesicles. Sja-mir-71a was detected in the serum extracellular vesicles of patients infected with *S. japonicum* [88], implying possible regulatory effects at distant sites. In this sense, distinct *S. japonicum* miRNAs were identified in extracellular vesicles from host serum, such asSja-let-7 and Sja-mir-190-5p, which were validated. Other non-validated miRNAs include Sja-mir-71a, Sja-mir-71b, Sja-mir-190-5p, Sja-let-7, and Sja-mir-36a [97]. Sja-bantam, Sja-mir-3479-3p, Sja-mir-10-5p, and Sja-mir-8185 were identified in the plasma of infected mice and rabbits [86]. Another miRNA, Sja-mir-3096, was detected in infected rabbit serum [86]. An independent report suggested the potential use of Sja-mir-277 and Sja-mir-3479-3p as biomarkers by analyzing the serum of infected mice, and found a correlation between the expression levels of these miRNAs and egg burden, as well as fibrosis in the liver [98]. A more extensive study distinguished 12 miRNAs in the serum of infected mice: Sja-mir-277, Sja-mir-3479-3p, Sja-mir-125a, Sja-mir-61, Sja-mir-2b-5p, Sja-mir-2162-3p, Sja-mir-36-3p, Sja-mir-3489, Sja-mir-3487, Sja-mir-2c-5p, Sja-mir-2a-3p, and Sja-mir-10. An analysis of miRNAs in the serum of infected patients showed that a combined analyses of two miRNAs, Sja-mir-2b-5p and Sja-mir-2c-5p, performed moderately well in discriminating between infected and uninfected individuals [99]. Thus, the identification of miRNAs in fluids such as serum or plasma could support the identification of affected individuals, in addition to the diagnostic procedures used. This strategy could help in the prevention of undesirable effects, such as granulomatous inflammation or fibrosis, which have been associated with tumorigenesis.

## 8. LncRNAs of *Schistosoma japonicum*

Information about the lncRNAs of *S. japonicum* is very limited. A bioinformatic analysis predicted 3033 potential lncRNAs encoded by *S. japonicum* using two RNA-sequencing libraries of adult male and female worms. An assessment of the sequence conservation between the lncRNAs of different *Schistosoma* species found a higher percentage of conservation between the lncRNAs of *S. mansoni* and *S. haematobium* than between the lncRNAs of *S. japonicum* and *S. haematobium* [100]. Recently, an analysis of 66 RNA sequencing libraries of cercariae, sporocysts, and schistosomula in adult males and females, predicted 12291 lncRNAs in *S. japonicum*, with 53.64% being intergenic lncRNAs (lincRNAs), 38.19% antisense lncRNAs, and 8.16% sense lncRNAs. Syntenic conservation in the lincRNAs of *S. japonicum* and *S. mansoni* was found, and could suggest functional conservation. Importantly, the landscape of lncRNA expression differed between mature females and immature females and males, suggesting the participation of lncRNAs in the regulation of sexual development. An in silico analysis suggested that lncRNAs could modulate the genic expression involved in different essential biological processes in the development of *S. japonicum* [101]. Further studies are necessary to identify the precise molecular mechanisms by which the lncRNAs regulate the biological functions of this parasite and to determine potential therapeutic applications.

## 9. miRNAs of *Clonorchis sinensis*

A total of 62512 miRNAs expressed in adult *C. sinensis* flukes were predicted, which were conserved with other species; additionally, 6 novel miRNAs were validated [102]. The analysis suggested that *C. sinensis* miRNAs such as Csi-mir-71, Csi-mir-277b, Csi-mir-71c, Csi-mir-215, and Csi-mir-36 could show higher expression levels [102]; this is interesting since their increased expression could be useful for diagnostic purposes; however, this has not yet been determined. Moreover, the functional roles of *C. sinensis* miRNAs in the life cycle of the parasite, in the infection mechanisms, and in the promotion of cancer are widely unknown.

A transcriptomic analysis of adult worms identified 51 miRNAs in *C. sinensis.* Among them, 27 miRNAs were conserved and 24 miRNAs were possibly species-specific [103]. However, reduced variation in the miRNAs of *C. sinensis* and high conservation have been reported [104]. Additionally, the genomic arrangement of some of these miRNAs has been determined. Two very conserved miRNA clusters (the mir-71a/2 cluster group and the mir-71b/2 cluster group) consisting of a set of four miRNAs were identified in *C. sinensis* [103]. Since the clusters could be expressed as a polycistronic transcript, it is possible that the miRNAs show comparable expression patterns [105]; however, the function of the miRNAs in *C. sinensis* clusters has not been determined.

Recently, the expression of miRNAs was analyzed in extracellular vesicles produced by adult *C. sinensis* worms. An increased expression of miRNAs such as Csi-mir-71a-5p, Csi-bantam, Csi-mir-61-3p, Csi-mir-10-5p, Csi-novel_mir11, and Csi-let-7a-5p was detected in extracellular vesicles [106]. Since these extracellular vesicles released by *C. sinensis* promoted macrophage M1/M2 polarization, biliary hyperplasia, fibrosis, and inflammation, it is possible that their miRNA cargo plays a role in host immune modulation [106], with a potential role in cancer promotion.

### Csi-let-7a-5p

**Figure 4:**
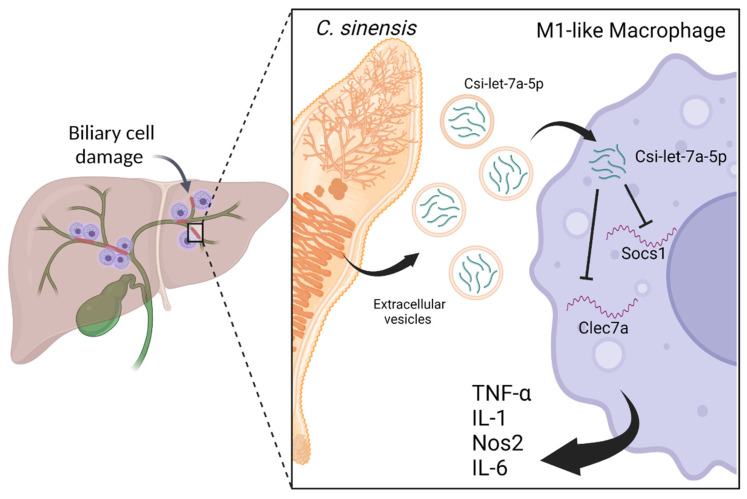
*C. sinensis* worms can release extracellular vesicles with miRNA cargo such as Csi-let-7a-5p. This miRNA is delivered to host macrophages, binds molecular targets inhibiting genic expression, and promotes polarization of these immune cells. Csi-let-7a-5p packaged in extracellular vesicles supports the accumulation of M1-like macrophages in the liver, which can lead to a proinflammatory microenvironment that has been connected to damage and proliferation of biliary cells. Created using BioRender.com.

Csi-let-7a-5p is the first miRNA of *C. sinensis* with a described function. This miRNA is loaded in extracellular vesicles, which can be released by worms [106]. Csi-let-7a-5p can be delivered to macrophages and exert regulatory functions by binding molecular targets [106] (Figure 4). Although the internalization of *C. sinensis* extracellular vesicles and the delivery of cargo molecules have been reported only in macrophages, it is possible that these events can take place in other host cellular types such as cholangiocytes, since the extracellular vesicles of *C. sinensis* can promote the proliferation of cholangiocytes and biliary injury [106]; however, this remains to be determined.

It has been proposed that Csi-let-7a-5p inside the macrophages binds the 3′-UTR region of Socs1 (a suppressor of cytokine signaling) and Clec7a (C-type lectin domain containing 7a), which provokes a reduction in their mRNA and protein expression, and this promotes the activation of the NF-κB signaling pathway. Ultimately, Csi-let-7a-5p induces polarization to M1-like macrophages, producing TNF-α, IL-6, IL-1b, Nos2 (nitric oxide synthase), CD80, and CD86, as well as accumulation in the liver [106]. It has been suggested that this proinflammatory effect contributes to inducing biliary injury (Figure 4) [106]. Inflammation related to *C. sinensis* infection has been connected to the promotion of cholangiocarcinoma [107]; thus, it is possible that Csi-let-7a-5p plays an important role in carcinogenesis, modulating the inflammatory microenvironment, and it can probably be a target for therapy or prevention.

## 10. miRNAs and lncRNAs of *Opisthorchis viverrini*

A genomic analysis predicted 178 miRNAs encoded by *O. viverrini* [108]. Additionally, an analysis of the transcriptomic profile using adult worms identified 17 miRNAs (as orthologs in *C. sinensis*) and 19 species-specific miRNAs as mostly intergenic miRNAs [103]. Interestingly, a bioinformatic analysis indicated a higher number of miRNAs (55) of *O. viverrini* being conserved among species such as *C. sinensis*. Additionally, it was suggested that these miRNAs could have similar expression patterns in the *O. viverrini* and *C. sinensis* species. Moreover, miRNA variation among species was limited, with the identification of two miRNAs (Ovi-mir-76 and Ovi-mir-new1) with different sequences that could be useful in distinguishing *O. viverrini* from other related organisms such as *C. sinensis* or *Opisthorchis felineus* [104].

Homology of the two miRNA clusters, the mir-71a/2 cluster group and the mir-71b/2 cluster group, encoded by *C. sinensis* were identified in *O. viverrine* [103], suggesting important roles in the functions of these parasites. Considering the information reported about *O. viverrini* miRNAs and the carcinogenic potential of this parasite, further studies should address aspects such as the biological functions, molecular targets, and roles in infection or in cancer of the miRNAs encoded by *O. viverrini*.

Finally, a genomic study using adult worms predicted 61 lncRNAs encoded *by O. viverrini* [108]; however, the expression of theses lncRNAs in different stages of development, possible molecular targets, functions in infection, packaging in extracellular vesicles, and their participation in promoting cancer have not been addressed.

## 11. Conclusions and Future Perspectives

miRNAs have been more widely studied than lncRNAs in carcinogenic parasites. Additionally, the reports analyzing miRNAs and lncRNAs in carcinogenic parasites are few in comparison to the available information regarding possible carcinogenic parasites (*S. japonicum*). A great number of miRNAs have been predicted to be encoded and expressed by these helminths involved in human cancers. The miRNAs of these parasites could be involved in carcinogenesis by modulating the biological functions of the pathogen and the host and by altering microenvironments prone to tumor growth (Figure 1). Additionally, miRNAs can be packed in parasite-released vesicles, delivered to host cells, and bind molecular targets, resulting in the modulation of processes associated with the promotion of cancer such as fibrosis, inflammation, and immune-cell polarization (Table 2). However, the molecular mechanisms and the functions of many of the miRNAs in these parasites are largely unknown.

LncRNAs have been expected to regulate essential biological processes and to be expressed by carcinogenic parasites, but studies analyzing the lncRNAs of *C. sinensis* have not yet been reported. Interestingly, lncRNAs were detected in extracellular vesicles released by helminths [109], but this has not been reported in carcinogenic or possibly carcinogenic parasites. It is important to determine the presence and the effects of lncRNA cargo in the extracellular vesicles produced by these parasites involved in cancer; moreover, it is important to establish whether lncRNAs represent another valuable layer of genic expression regulation implicated in the host–parasite interaction or in the promotion of cancer associated with infection.

The miRNAs of parasites involved in cancer have been detected in different fluids such as urine, serum, and plasma, and have potential as a diagnostic tool in infection and cancer. Studies on the circulating miRNAs of schistosomes have begun to demonstrate the potential reaches and possible difficulties in diagnostic applications [59,98,111]. In this sense, a plethora of candidate miRNAs encoded by carcinogenic parasites with potential in diagnostics should be analyzed in future studies. Moreover, the miRNA expression profiles are distinct in each developmental stage of the parasites associated with cancer, as is widely demonstrated in *S. japonicum*. The characterization of these profiles provides possible targets for the treatment and control of infection, as well as the prevention of conditions that could promote tumorigenesis. Interestingly, as reviewed, miRNAs encoded by *S. japonicum* can exert antitumoral effects by inhibiting fibrosis and the proliferation, growth, and migration of tumor cells. However, the miRNAs of this parasite can promote processes associated with cancer such as liver fibrosis and inflammation. Thus, the miRNAs of *S. japonicum* seem to contribute in dual ways when linked to cancer. The differences in the context of these studies could explain the apparent discrepancies.

The generation of biliary hyperplasia, fibrosis, microenvironments with high cytokine production, and the recruitment of inflammatory immune cells in chronic infection with *C. sinensis* have been linked to the promotion of cholangiocarcinoma [112,113]. Moreover, extracellular vesicles released by *C. sinensis* containing diverse molecules and miRNA cargo participate in the generation of these processes. An miRNA of this parasite is involved in the modulation of the host immune function and the promotion of inflammation [106]. Further studies will determine whether other miRNAs of this liver fluke are involved directly or indirectly in the promotion of cancer. Additionally, the functions of miRNAs or lncRNAs in the development, or throughout the life cycle, of *C. sinensis* have not been described experimentally. Studies in these directions could provide new targets for potential treatment.

Information about the expression and functions of miRNAs and lncRNAs from *O. viverrini* is very limited; thus, at the time, it is difficult to determine the contribution of these RNA regulators in the development of cholangiocarcinoma. Further studies should analyze the regulation of biological process across the lifespan of *O. viverrini* controlled by miRNAs and lncRNAs—as well as the possible participation of these RNA regulators in chronic injury to the biliary epithelial cells, inflammation, and host-cell proliferation—in order to identify potential therapeutic targets. This parasite can release extracellular vesicles with cargo molecules, which can be internalized by human cholangiocytes, thereby promoting the proliferation and production of proinflammatory cytokine IL-6. These vesicles have been described as having an important role in carcinogenesis [114]; however, to the best of our knowledge, an analysis of miRNA cargo in extracellular vesicles has not been carried out. It is possible to suggest that the extracellular vesicles of *O. viverrine* could transport miRNA cargo as many other helminths release extracellular vesicles containing miRNAs, and these exert biological effects on the cells of the host; however, these must be established. This could be useful for the identification of potential biomarkers. *Helicobacter pylori* has been associated with the promotion of extragastric cancers such as cholangiocarcinoma; additionally, a higher frequency of this bacterium was found in the bile of cholangiocarcinoma patients as compared with controls [115,116,117]. Interestingly, it has been suggested that *O. viverrini* serves as a reservoir host of *H. pylori* and is a vector for this carcinogenic bacterium in humans [118,119]. Thus, *O. viverrini* experimental infection in hamsters was found to increase the prevalence of *H*. *pylori* in bile samples, liver tissue, feces, and the rectal epithelium of infected hosts [116,118]. It was suggested that coinfection could promote opisthorchiasis-associated cholangiocarcinoma [119,120]. Together, these observations suggest that the microbiota of *O. viverrini* are important in the pathogenesis and carcinogenic potential of the parasite; whether the microbiota can modulate the miRNA or lncRNA expression profile of *O. viverrini* has not been determined. A recent study suggested that *O. viverrini* is not a reservoir of *H. pylori*, but that infection with *O. viverrini* or *C. sinensis* modifies the host’s microbiota by allowing the expansion of *H. pylori* already present in the host [116]. Whether the miRNAs or lncRNAs of oncogenic helminths participate in host microbiota alteration has not been reported.

miRNAs in different species share sequence similarities [68]. For example, Sja-mir-7-5p presents an identical seed sequence (2–8 nt in the 5′ region) to that of human Hsa-mir-7-5p. These miRNAs showed tumor-suppressor activity [94]. On the other hand, Sja-mir-125b induces the activation of stellate cells, inflammation, and macrophage polarization [84,91], which have been associated with tumor promotion. This miRNA from *S. japonicum* presents an identical seed sequence (2–8 nt in the 5′ region) to that of the human miRNA Hsa-miR-125a-5p. Interestingly, Hsa-miR-125a-5p may function as a powerful tumor promoter [121]. Thus, the miRNAs of helminths can share sequence similarities with human miRNAs and could show a similar capacity regarding the promotion or inhibition of tumor development. However, similar seed sequences between miRNAs do not indicate completely identical functions. Other mechanisms regulating gene expression, the availability of molecular targets, and the cellular context must be considered. For example, Hsa-miR-125a-5p can present oncogenic activity or tumor-suppressor functions in different types of cancer [121].

In addition to miRNAs and lncRNAs, interest in other regulator RNAs, such as circRNAs, has increased in recent years. CircRNAs are single-stranded closed RNAs that are very stable and resistant to degradation by RNases, with broad potential as biomarkers [122]. These molecules can function as miRNAs and protein sponges by regulating genic expression at different levels in health and disease. The key functions of circRNAs have been described in the context of cancer [122]. CircRNAs have been analyzed in distinct helminths and detected in parasite-derived extracellular vesicles, and their participation in diverse biological processes has been predicted [123,124,125]. However, the prediction, expression, and functional evaluation of circRNAs have not been addressed in carcinogenic and possibly carcinogenic helminth parasites. These analyses could bring to light potential new molecular mechanisms of regulation in the different biological functions of helminth parasites, as well as their possible applications in therapy or diagnostics in the context of cancer.

## Figures and Tables

**Table 1:** miRNAs encoded by *S. japonicum* with antitumoral activity.

miRNA	Host Molecular Target	Model	Biological Effect	Possible Seed Sequence	Ref ^‡^
Sja-mir-61	PGAM1	Liver tumor cells Xenograft tumor mouse model	Inhibition of cell migration Inhibition of cell growth	5′-GACUAGA-3′	[93]
Sja-mir-7-5p	SKP2	Liver tumor cells Xenograft tumor mouse model	Inhibition of cell proliferation Arrest of cell cycle Inhibition of cell migration	5′-UGGAAGA-3′	[94]
Sja-mir-71a	FZD4	Liver tumor cells Xenograft tumor mouse model	Inhibition of cell proliferation Arrest of cell cycle Inhibition of cell migration	5′-GAAAGAC-3′	[95]
Sja-mir-3096	PIK3C2A	Liver tumor cells Xenograft tumor mouse model	Inhibition of cell proliferation Inhibition of cell migration Arrest of cell cycle	5′-UGGACCA-3′	[96]
Sja-mir-3005; Sja-mir-3006; Sja-mir-3044; Sja-mir-7; Sja-mir-124	ND *	Liver tumor cells	Arrest of cell cycle	---------------	[95,96]

* Not determined; ^‡^ reference.

**Table 2:** Parasitic miRNAs possibly involved in cancer promotion.

miRNA	Host Molecular Target	Biological Effect	Possible Seed Sequence	Ref ^‡^
Sha-mir-71a	MAPK-3	ND * Found in urine of bladder cancer patients associated with infection	5′-GAAAGAC-3′	[56,59,110]
Sja-mir-1	SFRP1 ^¥^	Promotion of hepatic fibrosis and activation of HSC	5′-GGAAUGU-3′	[89]
Sja-mir-2162	TGFβ3 ^¥^	Promotion of hepatic fibrosis and activation of HSC	5′-UAUUAUGCA-3′	[84]
Sja-mir-125b, Sja-mir-219, Sja-mir-923, Sja-mir-3482 and Sja-mir-3480	ND *	Activation of HSC	---------	[84]
Sja-mir-125b	257 predicted putative targets PROS1 ^¥^	Promotion of inflammation Macrophage polarization	5′-UCCCUGAGA-3′	[64,68,84,91]
Sja-bantam	12 predicted putative targets FAM212B ^¥^ and CLMP ^¥^	Promotion of inflammation Macrophage polarization	5′-GAGAUCG-3′	[91]
Csi-let-7a-5p	SOCS1 ^¥^ and CLEC7A ^¥^	Promotion of inflammation Macrophage polarization Biliary injury	5′-GAGGUAG-3′	[106]

* Not determined; ^¥^ validated target; ^‡^ reference.
